# Adaptive Target Volume and Dosimetry in Image-Guided Radiotherapy for Cervical Cancer

**DOI:** 10.3390/jcm14103418

**Published:** 2025-05-14

**Authors:** Elena Manea, Beatrice Anghel, Anca Daniela Stanescu, Ana Maria Rata, Bogdan Gafton, Viorel Scripcariu

**Affiliations:** 1 Department of Surgery, “Gr. T. Popa” University of Medicine and Pharmacy, 700115 Iasi, Romania; manea.elena@d.umfiasi.ro (E.M.); gaftonbogdan@yahoo.com (B.G.); viorel.scripcariu@umfiasi.ro (V.S.); 2 Department of Radiotherapy, Regional Institute of Oncology, 700483 Iasi, Romania; 3 Department of Obstetrics and Gynecology, “Carol Davila” University of Medicine and Pharmacy, 050474 Bucharest, Romania; 4 Department of Radiotherapy, Sanador Oncology Centre, 010991 Bucharest, Romania; 5 Department of Obstetrics and Gynecology, “St. John” Emergency Hospital, Bucur Maternity, 040292 Bucharest, Romania; 6 Medical Clinical Department, Faculty of Medicine and Pharmacy, “Dunarea de Jos” University, 800010 Galati, Romania; anamariailie2418@gmail.com; 7 Department of Radiotherapy, “Sfantul Apostol Andrei” Emergency Clinical Hospital, 800578 Galati, Romania; 8 Department of Oncology, Regional Institute of Oncology, 700483 Iasi, Romania; 9 Department of Surgery, Regional Institute of Oncology, 700483 Iasi, Romania

**Keywords:** cervical cancer, image guided radiotherapy, target volume

## Abstract

**Background**: Cervical cancer treatment with advanced radiotherapy techniques benefits from image guidance, particularly when anatomical changes occur during therapy. This case emphasizes the need for adaptive radiotherapy when target volume shifts significantly. **Methods**: A 70-year-old woman with International Federation of Gynecology and Obstetrics (FIGO) IIIC2 9th edition cervical squamous cell carcinoma presented with a distended uterine cavity due to fluid accumulation. She underwent definitive chemoradiotherapy using Volumetric Modulated Arc Therapy (VMAT) and weekly cisplatin. **Results**: Daily Cone Beam Computed Tomography (CBCT) imaging revealed progressive uterine shrinkage as intrauterine fluid drained, significantly altering target volume and organ-at-risk (OAR) positioning. These changes necessitated two re-planning CT scans during external beam radiotherapy to maintain accurate dosing and avoid OAR toxicity. The patient completed treatment, including image-guided brachytherapy, without complications. Adaptive planning ensured adequate tumor coverage and minimized normal tissue exposure. **Conclusions**: This case highlights the critical role of daily CBCT in detecting anatomical changes during radiotherapy. Adaptive re-planning, though rarely required more than once, was essential here to preserve treatment accuracy. CBCT should be considered a standard verification tool in cervical cancer radiotherapy, particularly in cases involving intrauterine fluid.

## 1. Introduction

Cervical cancer is one of the most common malignancies affecting women, ranking as the fourth most prevalent cancer among this population. It is also the third leading cause of cancer-related deaths in women within developed countries. Notably, approximately 90% of cervical cancer fatalities occur in developing regions worldwide. These disparities are largely due to differences in access to human papillomavirus (HPV) screening and variations in HPV prevalence [1]. Nonetheless, cervical cancer continues to pose a significant public health concern even in high-income countries. When diagnosed early, cervical cancer is among the most treatable types of cancer, with high success rates for curative treatment. In more advanced stages, management shifts toward curative-intent or palliative approaches depending on the extent of disease [2].

Standard treatment for locally advanced cervical cancer typically involves a combination of external beam radiation therapy, concurrent chemotherapy, and a boost via brachytherapy. This multidisciplinary strategy has demonstrated favorable outcomes in terms of toxicity, survival, and local tumor control, particularly with the introduction of modern techniques that enhance precision and reduce adverse effects [3,4].

The evolution from conventional 2D radiotherapy to advanced modalities such as Intensity-Modulated Radiotherapy (IMRT) and Volumetric-Modulated Arc Therapy (VMAT) has led to improved dose distribution and conformity. These advancements allow for reduced radiation exposure to organs at risk (OARs), improving patient tolerance and decreasing treatment-related toxicities [3,4].

IMRT and VMAT are now considered the mainstays of modern radiotherapy for cervical cancer and are recommended as standard practices across institutions. However, the anatomical complexity of the pelvic region presents challenges due to organ motion and variability in target volumes and OARs. VMAT, a refined and dynamic form of IMRT, employs a continuously rotating gantry, variable dose rates, gantry speed and dynamic multileaf collimators to deliver treatment efficiently [5].

To manage anatomical uncertainties and minimize the risk of missing the target or overdosing surrounding organs, Image-Guided Radiotherapy (IGRT) plays a crucial role. IGRT allows for real-time assessment of organ and tumor position, improving both safety and treatment accuracy. VMAT technology supports comprehensive imaging before, during, and after treatment, significantly enhancing the quality and precision of cervical cancer management [6].

The most used IGRT modality is cone-beam computed tomography (CBCT), which utilizes megavoltage X-rays to generate in-room 3D imaging. CBCT allows for direct comparison between pre-treatment images and those used during radiotherapy planning. Routine CBCT monitoring facilitates verification of patient setup and positioning, while also providing essential data on target volume coverage and internal motion—both critical factors for delivering safe and effective radiotherapy [7].

Recent case reports have also underscored the impact of personalized, multimodal treatment approaches in complex or metastatic presentations of cervical cancer. For instance, integrating chemotherapy, radiotherapy, and immunotherapy has shown promising outcomes in aggressive histologies such as adenosquamous carcinoma, demonstrating the evolving landscape of cervical cancer treatment [8]. Recently, the Keynote report shows supporting evidence for addition of immunotherapy in cervical cancer treatment [9].

This case report aims to highlight the clinical challenges and adaptive radiotherapy strategies in a patient with locally advanced cervical squamous cell carcinoma complicated by significant uterine fluid accumulation. It emphasizes the critical role of daily CBCT imaging in identifying anatomical changes during treatment and guiding timely re-planning to optimize tumor coverage and spare organs at risk.

## 2. Case Presentation

We report the case of a 70-year-old woman clinically diagnosed with stage IIIC2 cervical squamous cell carcinoma, according to FIGO classification (cT2bN1M0), based on imaging and histopathological findings.

The patient initially presented to the Gynecology Department with vaginal bleeding. A clinical examination revealed a malignant cervical mass, and a biopsy was performed at the lesion site. Histological analysis confirmed a poorly differentiated (G3) squamous cell carcinoma of the cervix.

Abdominal and pelvic magnetic resonance imaging (MRI) with intravenous contrast showed an enlarged uterus measuring 106 × 84 × 135 mm (Anterior Posterior (AP) × Transversal (T) × Cranio-Caudal (CC)), containing a fluid-filled uterine cavity with a thickness of 56 mm. The cervix was infiltrated by a centrally located lesion measuring 27 × 38 × 23 mm (AP × T × CC), causing cervical stenosis, with bilateral parametrial invasion and involvement of the lower portion of the left vaginal wall.

Multiple lymph node enlargements were observed, including lumbo-aortic nodes (largest: 13 × 10 mm), left internal iliac (8 × 10 mm), left obturator (15 × 12 mm), and left external iliac (8 × 9 mm). A stenotic lesion at the cervical level, along with a real fluid-filled cavity (hydrometra), was also noted. Staging was completed with a contrast-enhanced thoracic CT, which revealed no evidence of distant metastases. Routine blood tests were normal.

Based on clinical, imaging, and histopathological findings, the disease was staged, and the treatment plan included concurrent chemoradiotherapy followed by image-guided adaptive brachytherapy.

At the radiotherapy department, the patient underwent a planning CT scan of the abdomen and pelvis to prepare for treatment. Simulation was conducted in the supine position using the “arm shuttle” platform, which is typically employed for cases involving para-aortic lymph node irradiation. The CT scan was acquired with a 3 mm slice thickness, using intravenous contrast to aid in vascular delineation. Both bladder and rectal preparation protocols were observed during simulation and daily treatments, and the patient was instructed to adhere strictly to these protocols.

For bladder filling, the patient drank 500 mL of water one hour prior to both simulation and each treatment session. Daily imaging was planned using cone-beam CT (CBCT) verification. Clinical target volumes (CTV) were delineated according to guidelines from the Radiation Therapy Oncology Group (RTOG), the GYN IMRT Consortium, and the Japan Clinical Oncology Group [10,11]. Planning target volumes (PTV) were subsequently created based on each CTV (according to EMBRACE II protocol basic IGRT). Per protocol, at least 95% of each PTV was to receive 100% of the prescribed dose.

The patient began definitive external beam radiotherapy using the VMAT technique with Simultaneous Integrated Boost (SIB), delivered on a Clinac iX15 Linear Accelerator, manufactured by Varian Medical Systems. The prescribed doses were as follows:

- An amount of 45 Gy in 25 fractions (1.8 Gy/fraction) to the tumor, cervix, uterus, vagina, parametria, and nodal regions including bilateral common, internal, external, obturator, superior presacral, and para-aortic lymph nodes.
- An amount of 55 Gy in 25 fractions (2.2 Gy/fraction) to pelvic lymph node metastases.
- An amount of 60 Gy in 25 fractions (2.4 Gy/fraction) to para-aortic lymph nodes.

Concurrently, the patient received four cycles of weekly chemotherapy with cisplatin at a dose of 40 mg/m^2^.

After completion of chemoradiotherapy, the patient proceeded to image-guided brachytherapy D90 HR_CTV 7Gy four treatments every other day. Due to residual disease, interstitial brachytherapy was required. Target delineation and treatment planning were performed in accordance with the recommendations of the Groupe Européen de Curiethérapie and the European Society for Radiotherapy and Oncology (GEC-ESTRO) [12,13].

Throughout radiotherapy, daily CBCT verification was conducted. Imaging revealed changes in the irradiated volume due to a reduction in uterine size following the drainage of intrauterine fluid. This anatomical shift necessitated a new planning CT scan. Following reimaging, both target volumes and OARs were recontoured.

The medical physicist created a new treatment plan using the same technique, accounting for the number of fractions already delivered. The patient continued treatment under the updated plan, with enhanced attention to CBCT verification.

Due to continued anatomical changes in the uterus following fluid removal, the patient required two re-plannings during the course of treatment. A new treatment plan was generated after each CT scan. Despite these adaptations, the patient completed treatment without complications.

**Figure 1:**
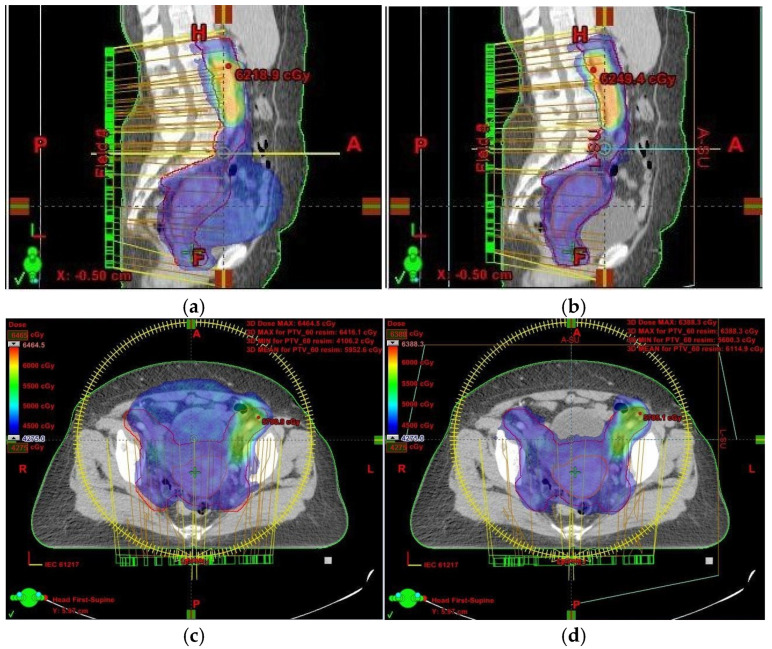
(**a**–**d**) CT scan treatment planning for the initial and rescan plan—axial and sagittal views: comparison of the 95% isodose curve from the PTV (blue)—**left** (**a**,**c**): initial plan; **right** (**b**,**d**): rescan plan. PTV margin is indicated in red. On the left, the original irradiated volume (blue) significantly exceeds the updated PTV post-fluid removal, leading to unnecessary exposure of healthy tissue.

**Figure 2:**
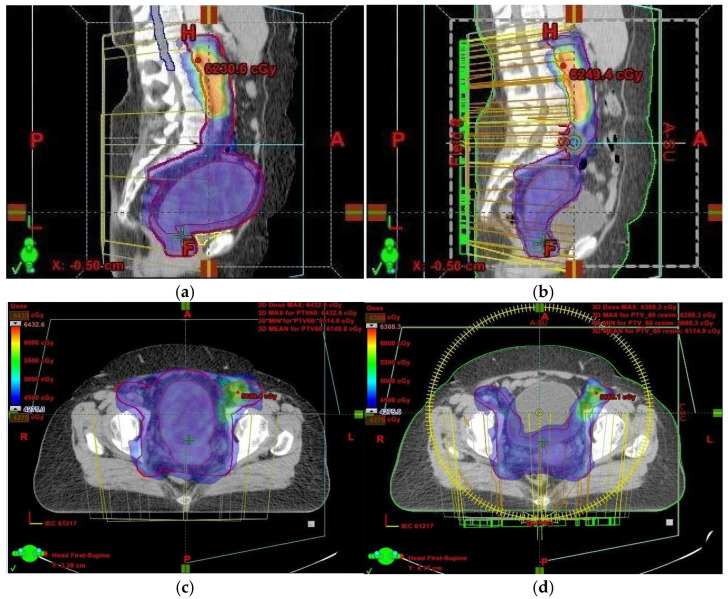
(**a**–**d**) CT scan treatment planning—axial and sagittal views: comparison of the 95% isodose curve from the PTV (blue)—**left**: initial plan; **right**: rescan plan. PTV margin is shown in red. These images illustrate the substantial differences in volume and dose distribution between the two plans.

Had we relied solely on kilovoltage (KV) imaging instead of CBCT, the irradiated volume would have remained unchanged, potentially resulting in overdosing of healthy tissues and missing the target volume altogether (Figure 1 and Figure 2). This reinforces the necessity of routine CBCT verification when employing VMAT, especially in cervical cancer cases.

In light of these findings, we analyzed the impact of target volume changes and their effect on OARs. A comparison of the first and final treatment plans revealed that the CTV1, which included the uterus, decreased from 561.44 cm^3^ to 155.33 cm^3^. Similarly, the summed PTV (PTVsum) was reduced from 2165.58 cm^3^ in the initial plan to 1577.25 cm^3^ in the final plan. The OARs assessed included the bladder, rectum, sigmoid colon, and bowel bag. Their respective volumes were as follows:

- Bladder: 83.28 cm^3^;
- Rectum: 33.37 cm^3^;
- Sigmoid: 33.19 cm^3^;
- Bowel bag: 2591.62 cm^3^.

Table 1 provides a clear quantitative overview, supporting the case for adaptive re-planning in anatomically dynamic pelvic tumors.

The patient was followed clinically, with imaging performed every 3 months for the first 2 years. She presented complete response without side effects.

## 3. Discussion

Image-Guided Radiotherapy is a modern and essential tool in the delivery of radiotherapy, offering significant dosimetric advantages when used in conjunction with techniques like IMRT and VMAT. One of the key challenges in pelvic irradiation, particularly in cervical cancer, is organ and tumor motion, which introduces uncertainty and compromises treatment accuracy. IGRT plays a pivotal role in overcoming this by enabling real-time verification and adjustment. The primary benefits of this approach include dose escalation and reduced toxicity. Identifying target misses and preventing overdose to OARs are critical components of safe and effective VMAT treatment.

This case highlights a specific challenge encountered during cervical cancer treatment—the impact of fluid retention in the uterine cavity on target volume delineation and adjacent normal tissues. The IGRT system, specifically CBCT, enables pre-treatment image verification. CBCT images are overlaid with the original planning CT to visualize the tumor volume and OARs, allowing for adjustments in real time. Tumor volume alterations may result from a variety of physiological and pathological changes during treatment [6,14]. In this case, the patient presented with significant uterine enlargement due to intrauterine fluid accumulation, secondary to cervical stenosis. Gradual drainage of the fluid occurred during treatment, leading to a substantial reduction in uterine volume and necessitating treatment re-planning. These volumetric changes were identified on daily CBCT scans, which revealed considerable differences and led to the creation of a new treatment plan. Two re-planning CT scans were ultimately required to adapt to the changing anatomy.

The most pronounced change was observed in the bladder. This is likely due to compression by the enlarged uterus in the initial CT scan, which resolved following fluid drainage, allowing the bladder to return to its typical filling capacity.

Technological advances have made the real-time identification of target volumes possible. VMAT offers precise dose conformity to the target volume while sparing adjacent healthy tissue. However, due to ongoing anatomical changes—particularly in the CTV—there remains a risk of the target volume moving out of the high-dose region, resulting in underdosing.

To address this issue, the International Commission on Radiation Units and Measurements (ICRU) Report 62 introduced the concept of a safety margin. ITV represents a new definition for motion of CTV in the patient, but it does not account for setup uncertainties. The PTV, accounts for both internal organ motion and setup errors [15]. The decision to define PTV margins involves balancing the risk of underdosing the tumor against the potential for complications in OARs.

Determining appropriate PTV margins is influenced by daily variations in target volume and OAR position [16,17]. Bladder filling significantly affects target coverage. IGRT is typically adapted during the planning stage, but other factors such as rectal filling, bloating, and bladder volume fluctuations also impact dose delivery. These factors can largely be managed by strict adherence to protocol [7,18]. Tumor regression is another dynamic variable. However, previous studies suggest that while tumor shrinkage is common, its impact on adapting EBRT remains limited [6].

Buchali et al. demonstrated that bladder filling influences uterine position but does not significantly affect the anterior–posterior positioning of the cervix [19]. Furthermore, the image quality of CBCT plays a vital role in accurately detecting changes that could impact the target volume.

In our case, although the target volume remained within the prescribed isodose region, the issue arose because OARs were inadvertently included within the irradiated volume (Figure 1). This was a result of uterine volume reduction following fluid elimination. Since the dose remained unchanged while the target volume decreased, more normal tissue was exposed to high-dose radiation. To mitigate this, we anticipated potential anatomical changes and implemented daily CBCT imaging. While re-planning during cervical cancer treatment is relatively uncommon, it is crucial to detect significant anatomical shifts early—something only achievable through routine CBCT setup monitoring.

In the context of locally advanced cervical cancer, the necessity for treatment re-planning due to significant anatomical changes is uncommon, and instances requiring multiple adaptations within a single treatment course are particularly rare. For example, a recent case study highlighted the use of adaptive radiotherapy to manage substantial daily uterine movement, necessitating re-optimization of each treatment fraction to maintain optimal tumor coverage and minimize exposure to OARs. Similarly, a dosimetric study evaluated a daily online adaptive protocol using CBCT-based platforms, demonstrating improved OAR dose metrics and suggesting that daily adaptation with reduced margins could decrease toxicity while maintaining target coverage [20,21]. Our case is distinctive in that it involved two separate re-planning instances prompted by the progressive resolution of intrauterine fluid accumulation. This scenario underscores the dynamic nature of tumor and organ anatomy during treatment and the critical importance of vigilant monitoring. While many centers may not routinely implement multiple adaptive strategies within a single treatment course, our experience suggests that such flexibility can be essential for certain patients to ensure both the efficacy and safety of radiotherapy.

The recent literature also highlights the broader shift toward individualized treatment strategies in gynecologic oncology. As reviewed by Hamoud et al. [22], the integration of immunotherapy and precision oncology is reshaping the management of uterine cancers, with emphasis on tailoring therapy to tumor biology, immune microenvironment, and patient-specific factors [9]. While our case centers on radiotherapy adaptation, it aligns with this emerging paradigm of dynamic, patient-adaptive cancer care—reinforcing that responsiveness to real-time anatomical or biological changes is key to improving outcomes.

Given the challenges presented by anatomical changes such as uterine cavity distension, it is worth considering whether daily CBCT imaging when treated with IMRT/VMAT should become a standard practice for all cervical cancer patients exhibiting such conditions. Routine daily imaging could facilitate the timely detection of significant anatomical alterations, allowing for prompt intervention and adaptation of treatment plans. This approach has the potential to enhance tumor targeting precision and reduce the risk of unnecessary radiation exposure to OARs, thereby improving overall treatment outcomes.

In the long term, adaptive planning can lead to more precise and effective treatments, fewer side effects, and better integration into everyday clinical practice. However, it also brings challenges, such as the need for more resources and changes to workflow. The repeated imaging and re-planning involved in adaptive radiotherapy produce a large amount of useful data. Over time, these data can help create predictive tools to identify which patients will benefit most, leading to more personalized and efficient care.

Frequent re-planning in adaptive radiotherapy, while clinically beneficial, can strain clinical resources, extend patient treatment times, and pose challenges for institutions lacking advanced technology and trained staff. To avoid overwhelming workflows, its use should be guided by clear protocols that balance clinical benefits with practical limitations.

AI and automated planning systems could greatly improve the efficiency of adaptive radiotherapy by speeding up contouring, planning, and identifying when re-planning is truly needed. As these tools advance, they may help reduce clinician workload and expand access to high-quality, consistent treatment, though expert oversight will still be crucial.

In conclusion, this case highlights the value of adaptive radiotherapy and supports the growing trend toward precision cancer treatment. Using daily imaging and plan adjustments can improve outcomes in cancers where anatomy changes during treatment and should be considered for inclusion in future treatment guidelines. This case report describes the experience of a single patient, which limits the ability to generalize the findings to a broader patient population.

## 4. Conclusions

Deploying the IGRT system to treat cervical cancer is difficult due to the complexity of target and OAR motions. In the era of IGRT, CBCT allows the imaging verification of patient position and preparation, as well as the changes in the size and shape of the tumor, with the possibility of real-time error correction, ensuring optimal treatment quality. Through this case, we highlighted the importance of modern radiotherapy techniques in cervical cancer, and this should represent a standard in all institutions. The IGRT system allows treatment to be adjusted based on changes in anatomy, thus ensuring that treatment is administered correctly. This case of locally advanced cervical cancer has shown an innovative approach of treating with high conformal radiotherapy in reductions in treated volumes.

## Figures and Tables

**Table 1:** Quantitative comparison of pre- and post-adaptive radiotherapy plans.

Parameter	Initial Plan	Final Plan	% Change
CTV1 Volume (cm^3^)	561.44	155.33	↓ 72.3%
PTVsum Volume (cm^3^)	2165.58	1577.25	↓ 27.2%
HR-CTV D90 (* EQD2, Gy)	83.5	87.4	↑ 4.7%
Bladder D2cc (EQD2, Gy)	85.4	77.1	↓ 9.7%
Rectum D2cc (EQD2, Gy)	74.3	68.2	↓ 8.2%
Sigmoid D2cc (EQD2, Gy)	70.5	65.0	↓ 7.8%
Bowel Bag V45 (cm^3^)	190	130	↓ 31.6%

* EQD2 values calculated assuming α/β = 3 Gy for OARs and α/β = 10 Gy for tumor.

## Data Availability

Data are available on request at the corresponding authors.
